# Pharmacological inhibition of G9a/GLP restores cognition and reduces oxidative stress, neuroinflammation and β-Amyloid plaques in an early-onset Alzheimer’s disease mouse model

**DOI:** 10.18632/aging.102558

**Published:** 2019-12-04

**Authors:** Christian Griñán-Ferré, Laura Marsal-García, Aina Bellver-Sanchis, Shukkoor Muhammed Kondengaden, Ravi Chakra Turga, Santiago Vázquez, Mercè Pallàs

**Affiliations:** 1 Pharmacology Section, Department of Pharmacology, Toxicology, and Therapeutic Chemistry, Faculty of Pharmacy and Food Sciences, Institute of Neuroscience, University of Barcelona (NeuroUB), Barcelona 08028, Spain; 2 Chemistry Department and Center for Diagnostics and Therapeutics, Georgia State University, Atlanta, GA 30303, USA; 3 Department of Biology, Georgia State University, Atlanta, GA 30303, USA; 4 Laboratori de Química Farmacèutica (Unitat Associada al CSIC), Department de Farmacologia, Toxicologia i Química Terapèutica, Facultat de Farmàcia i Ciències de l’Alimentació, and Institute of Biomedicine (IBUB), Universitat de Barcelona, Barcelona E-08028, Spain

**Keywords:** G9a/GLP, epigenetics, neuroinflammation, synaptic plasticity, β-amyloid plaques

## Abstract

The implication of epigenetic mechanisms in Alzheimer’s disease (AD) has been demonstrated in several studies. UNC0642, a specific and potent inhibitor of methyltransferase activity G9a/GLP (G9a-like) complex, was evaluated in the 5XFAD mouse model. UNC0642 treatment rescued 5XFAD cognition impairment, reduced DNA-methylation (5-mC), increased hydroxymethylation (5-hmC), and decreased the di-methylation of lysine 9 of histone H3 (H3K9me2) levels in the hippocampus. Increases in the Nuclear Factor erythroid-2-Related Factor 2 (NRF2), *Heme oxygenase decycling 1* (*Hmox1)* gene expression, and diminution in Reactive Oxygen Species (ROS) were also reported. Moreover, neuroinflammatory markers, such as *Interleukin 6* (*Il-6), Tumor necrosis factor-alpha (Tnf-α*) gene expression, and Glial fibrillary acidic protein (GFAP) immunofluorescence were reduced by UNC0642 treatment. An increase in *Nerve growth factor (Ngf), Nerve growth factor inducible (Vgf)* gene expression, Brain-derived neurotrophic factor (BDNF), and Synaptophysin (SYN) were found after UNC0642 treatment. Importantly, a reduction in β-amyloid plaques was also observed. In conclusion, our work demonstrates that the inhibition of the G9a/GLP complex by UNC0642 delivered significant neuroprotective effects in 5XFAD mice, point out G9a/GLP as a new target for AD.

## INTRODUCTION

Alzheimer’s disease (AD), a progressive neurodegenerative disease, is the main cause of dementia and its most significant factor is advanced age [1]. AD is characterized by the presence of extracellular senile or β-amyloid (Aβ) plaques and, intraneuronal neurofibrillary tangles (NFTs) formed by hyperphosphorylated tau aggregates; and by neuronal death [2, 3]. High levels of Aβ induce synaptic dysfunction and at the end loss of synapses [4–6]. Besides, Aβ plaques could contribute to NFTs, neuroinflammation, oxidative damage, and changes in chromatin structure [7, 8].

Some critical events known in AD are oxidative stress (OS), synaptic loss and glial responses as neuro-inflammation [9]. Regarding OS, it may damage the nervous system and lead to synaptic dysfunction [10] and is relevant in neurodegenerative diseases as AD [11]. Furthermore, in the inflammation process, some cytokines produce neuronal damage and a higher expression and changes in Amyloid precursor protein (APP) processing of [12]. Moreover, Tumor Necrosis Factor-alpha (TNF-α) and Interleukin 6 (IL-6) have been detected to aggravate both Aβ and tau pathologies in AD [13].

The regulation of epigenetic mechanisms plays a key role in human health, brain development, and function, being implicated in neurological disorders. Various mechanisms, such as DNA methylation (5-mC), hydroxymethylation (5-hmC), histone modifications, and regulation of the non-coding RNA, regulate the accessibility of chromatin to transcription factors and, therefore, these modifications are implicated in the modulation of DNA replication, transcription, and repair [14]. Consequently, its dysregulation is closely related to cognitive decline in aging [15] and transcriptional changes in various neurogenerative diseases such as AD, Huntington’s disease (HD) and Amyotrophic lateral sclerosis (ALS) [14, 16]. By one hand, histone modifications found in aging affect the transcription of different genes as the Brain-derived neurotrophic factor (BDNF) involved in learning and memory. In fact, the more BDNF levels, the less cognitive decline in aging, being widely accepted as a neuroprotective inductor [14, 17]. On the other hand, the role of epigenetics in the regulation of the mechanisms mentioned above: OS [18], neuroinflammation [19], and synaptic plasticity [20] are described elsewhere.

Histone epigenetic modifications include acetylation, methylation, phosphorylation, and ubiquitination [21]. Growing evidence suggests that histone methyltransferases act as a crucial regulator in human diseases [22]. G9a and G9a-like (GLP) protein are lysine methyltransferases that form a heterodimeric complex able to mono- and di-methylate lysine 9 of histone H3 (H3K9me1 and H3K9me2) of the N-terminal tail. Those epigenetic modifications lead to transcription repression. The G9a/GLP complex plays a role in learning and memory because its inhibition participates in the maintenance of long-term potentiation (LTP), long-term depression (LTD) [23], and also increases *Bdnf* gene expression [17].

Given the evidence demonstrating the implication of the G9a/GLP complex in different human diseases, it has emerged as a promising pharmacological target, and several small-molecules have been designed to inhibit these enzymes [24]. Optimization of these molecules lead to UNC0642, a compound with IC_50_ < 2.5 nM and optimized pharmacokinetics (PK) [24, 25]. This inhibitor of G9a/GLP was the first *in vivo* chemical probe with high potency in reducing H3K9me2 levels, and low cell toxicity (EC_50_ > 3,000 nM). Regarding the *in vivo* PK properties, administration of 5 mg/kg was shown to have a maximum concentration (Cmax) in plasma of 947 ng/mL, 68 ng/mL in the brain and was well tolerated [26]. Furthermore, 5mg/Kg dose is sufficient to inhibit G9a/GLP activity in adult mice [27].

The 5XFAD is a suitable transgenic mouse model of Early-Onset AD (EOAD), developing AD hallmarks as Aβ accumulation, plaques and cognitive impairment as early as 4-month-old [28–30]. Likewise, the 5XFAD model shows synaptic degeneration [31], mitochondrial dysfunction [32], increased OS [33], and microglial activation [34]. Additionally, epigenetic aberrations in the 5XFAD model were also described [35]. Of note, the critical role of epigenetics in 5XFAD was revealed in a recent study, including a correlation among cognitive impairment, Aβ pathology, and epigenetic modifications [33].

The present work aimed to evaluate the beneficial effects of the pharmacological inhibition activity of G9a/GLP with UNC0642 in 5XFAD mice, evaluating epigenetic changes, cognitive improvement, and the influence of the G9a/GLP complex inhibition in amyloid pathology, OS, neuroinflammation, and neuronal plasticity.

## RESULTS

### Beneficial effects on behaviour and cognition induced by UNC0642 in 5XFAD mice

**Figure 1:**
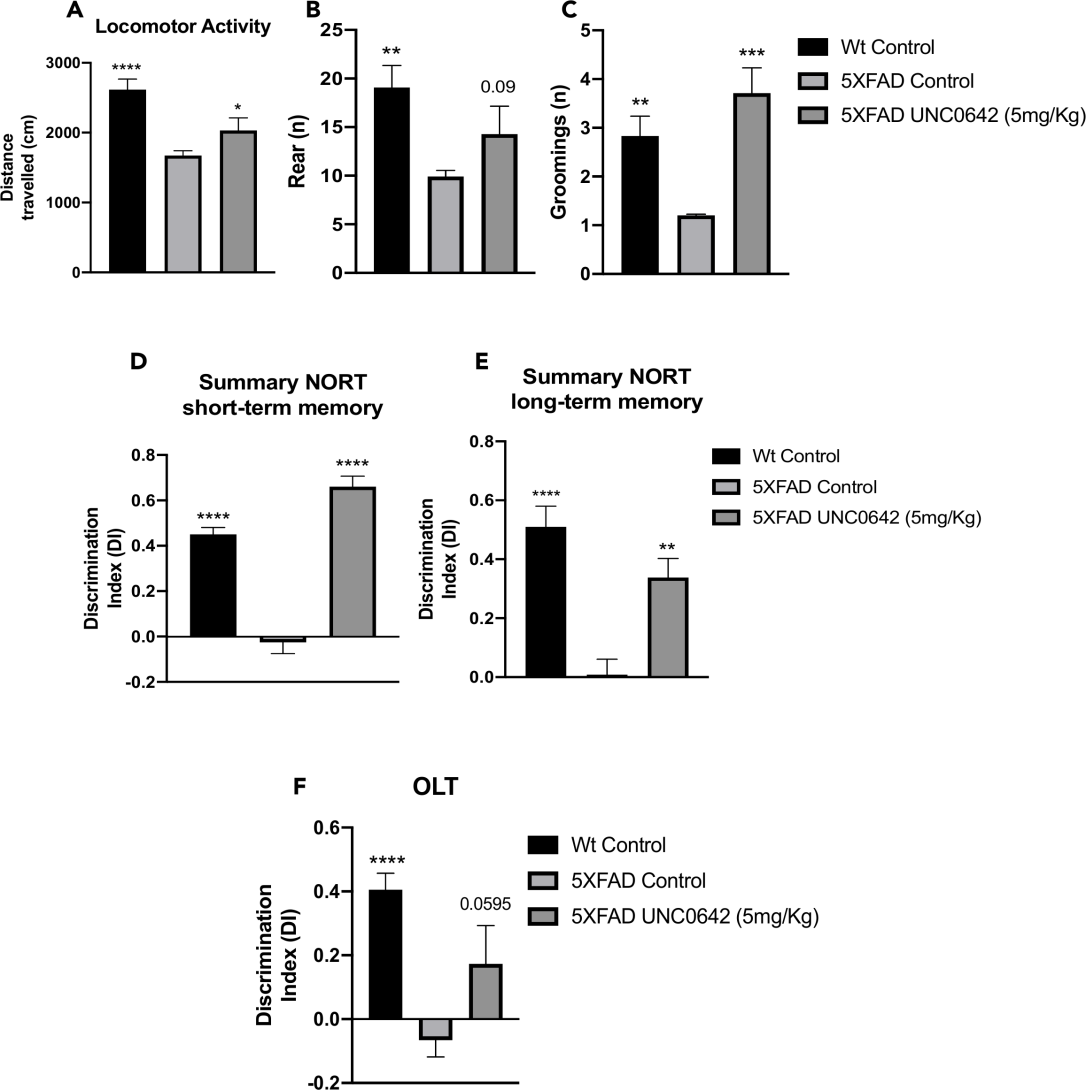
Results of the OFT, DI of the NORT, and DI of the OLT in male mice at 8-month-old Wt Control, 5XFAD Control, and 5XFAD treated with UNC0642 (5mg/Kg) mice groups. Locomotor Activity (**A**), Rearings (**B**), and Groomings (**C**). For NORT: Summary of the short-term memory (**D**), and long-term memory (**E**). Summary of DI (**F**). Values represented are the mean ± Standard error of the mean (SEM); (n = 27 (Wt Control n = 10, 5XFAD Control = 10, 5XFAD UNC0642 n = 7)). *p<0.05; **p<0.01; ***p<0.001; ****p<0.0001.

5XFAD treated with UNC0642 restored the locomotor activity in comparison with the 5XFAD Control group (Figure 1A). Likewise, an increase in vertical activity, quantified by the number of rearings, compared to the 5XFAD Control group was found (Figure 1B). By last, a significant increase in grooming in 5XFAD treated with UNC0642 in comparison with 5XFAD Control group was found (Figure 1C). All these parameters were significantly altered in 5XFAD Control in comparison with Wild-type (Wt) Control (Figure 1A–1C). Additional parameters measured in the Open Field Test (OFT) are presented in Table 1.

**Table 1:** Parameters measured in the Open Field Test (OFT).

	**Wt Control**	**5XFAD Control**	**5XFAD UNC0642 (5mg/Kg)**
**Locomotor activity (cm)**	2,614.55 ± 153.22****	1,672.36 ± 68.84	2,030.46 ± 180.96*
**Distance in zone-Center (cm)**	102.09 ± 11.20	87.64 ± 8.21	92.74 ± 8.83
**Distance in zone-Periphery (com)**	1,983.63 ± 143.41**	1,450.05 ± 133.56	1,623.83 ± 175.20
**Rearings (n)**	19.08 ± 2.26**	9.9 ± 0.66	14.29 ± 2.87^0.09^
**Grommings (n)**	2.83 ± 0.41**	1.2 ± 0.25	3.71 ± 0.52***
**Defecations (n)**	2.17 ± 0.44**	0.80 ± 0.20	0.86 ± 0.55
**Urinations (n)**	0.33 ± 0.26	0.0 ± 0.00	0.0 ± 0.00

(n): number of events. Results are expressed as a mean ± Standard error of the mean (SEM). *p <0.05; **p <0.01; ***p<0.001; ****p <0.0001 vs 5XFAD Control.

On the other hand, cognition was measured by the Novel Object Recognition Test (NORT) and Object Location Test (OLT) tests. NORT analysis demonstrated that 5XFAD treated with UNC0642 mice exhibited significantly reduced cognitive deficits in both short- and long-term memory presented by 5XFAD Control mice group (Figure 1D and 1E). Moreover, we confirmed the cognitive impairment of the 5XFAD mouse model in both short- and long-term memories compared to the Wt Control mice group (Figure 1D and 1E). Regarding OLT evaluation, a higher Discrimination Index (DI) value in 5XFAD treated with UNC0642 compared to the 5XFAD Control group was found (Figure 1F), demonstrating the beneficial effects on cognition after pharmacological inhibition of G9a/GLP in 5XFAD, restoring it to Wt phenotype.

### UNC0642 treatment decreased 5-mC, increased 5-hmC and reduced the hippocampus H3K9me2 levels in 5XFAD mice

**Figure 2:**
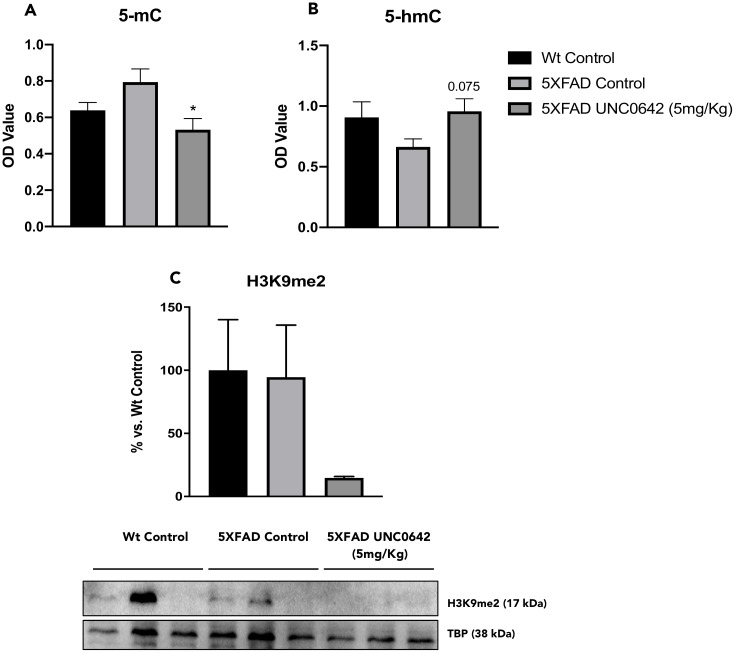
Global 5-methylated (**A**), and 5-hydroxymethylated cytosine levels (**B**) in the hippocampus from different mice groups. Representative Western Blot, and quantification for H3K9me2 (**C**). Values in bar graphs are adjusted to 100% for protein levels of the Wt Control. Values represented are mean ± Standard error of the mean (SEM); (n = 12 (Wt Control n = 4, 5XFAD Control = 4, 5XFAD UNC0642 n = 4)). *p<0.05.

We first evaluated the global levels of 5-mC and 5-hmC in DNA samples. We found a significant reduction in 5-mC levels in 5XFAD treated with UNC0642 in comparison with the 5XFAD Control mice group. In parallel, 5-hmC levels were increased in 5XFAD treated with UNC0642 compared to the 5XFAD Control (Figure 2A and 2B). Finally, we measured the effectiveness of UNC0642 inhibiting G9a/GLP in the brain through the evaluation of H3K9me2 by Western Blotting (WB). UNC0642 treatment reduced H3K9me2 levels compared to the 5XFAD Control, and no changes were found between Control mice groups (Figure 2C).

### UNC0642 treatment activated the NRF2 pathway, which leads to a reduction in OS levels

**Figure 3:**
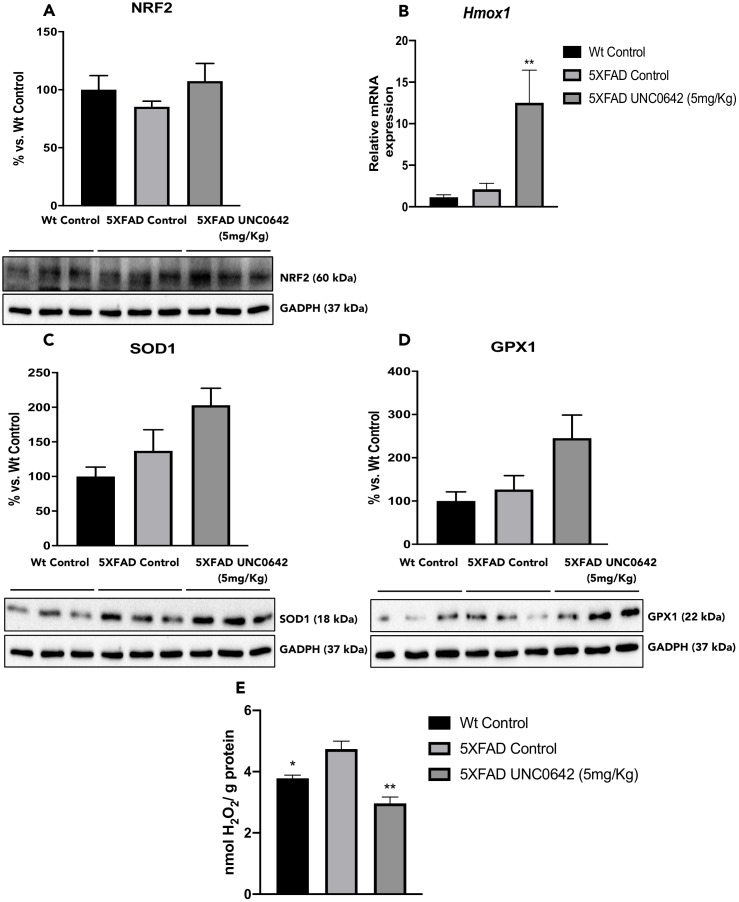
Representative WB, and quantification for NRF2 (**A**), SOD1 (**C**), and GPX1 (**D**). Representative gene expression for *Hmox1* (**B**). Representative OS measured as hydrogen peroxide concentration in homogenates of the hippocampus tissue (**E**). Values in bar graphs are adjusted to 100% for protein levels of the Wt Control. Gene expression levels were determined by real-time PCR. Values represented are mean ± Standard error of the mean (SEM); (n = 3-6 for each group). *p<0.05; **p<0.01.

To address the question of whether UNC0642 treatment reduces OS by upregulating the Nuclear factor erythroid-2-related factor 2 (NRF2) pathway, we evaluated protein levels of the transcription factor NRF2. We found increased in NRF2 protein levels in 5XFAD treated with UNC0642 compared to the 5XFAD Control mice group (Figure 3A). In parallel fashion, the expression of NRF2 targets was increased in animals treated with UNC0642. Specifically, *Heme oxygenase decycling 1* (*Hmox1)* gene expression, Superoxide dismutase 1 (SOD1) and Glutathione peroxidase 1 (GPX1) protein levels were increased in 5XFAD treated with UNC0642 in comparison with the 5XFAD Control group, being only significant for the *Hmox1* gene expression (Figure 3B–3D). Furthermore, no changes were found between both Control mice groups, demonstrating the activation of the NRF2 pathway through the inhibition of the G9a/GLP.

By last, evaluation of hydrogen peroxide (H_2_O_2_) levels in homogenates of the hippocampus tissue demonstrated a significant decrease in Reactive Oxygen Species (ROS) levels in 5xFAD treated with UNC0642 compared to the 5XFAD Control (Figure 3E). Likewise, a significant reduction in ROS levels in Wt Control in comparison to the 5XFAD Control was found, confirming the pathogenic phenotype (Figure 3E).

### Reduction of neuroinflammation after treatment with UNC0642 in 5XFAD mice

**Figure 4:**
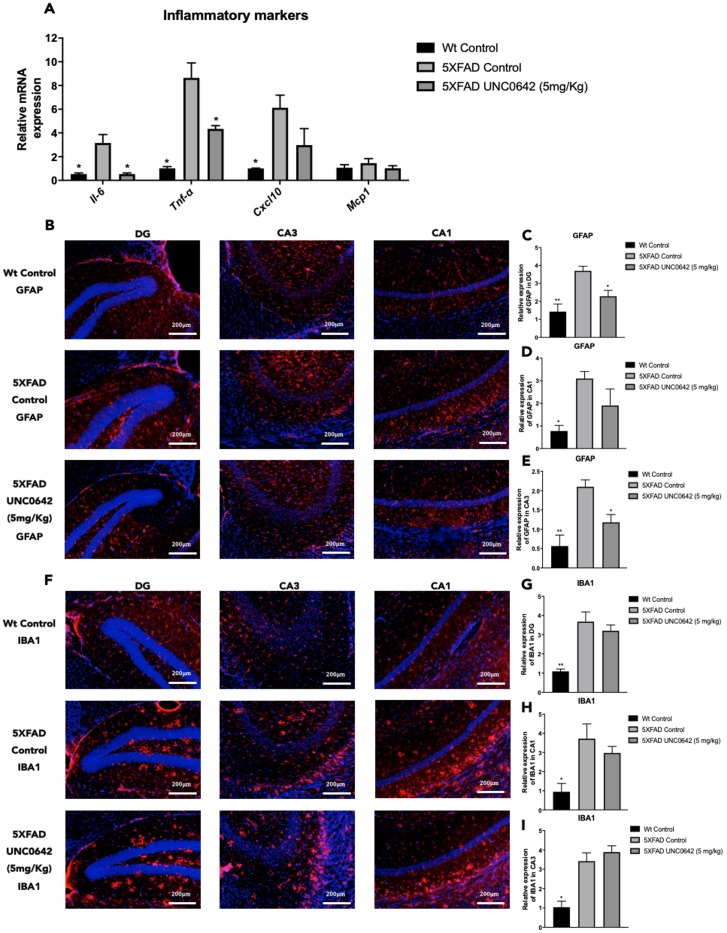
Representative gene expression of inflammatory markers for *Il-6, Tnf-α, Cxcl10,* and *Mcp1* (**A**). Representative images for GFAP (**B**) and IBA1 immunostaining (**F**) and quantifications for GFAP on the bar chart (**C**, **E**), and for IBA1 (**G**–**I**). Gene expression levels were determined by real-time PCR. Values represented are mean ± Standard error of the mean (SEM); (n = 4-6 for each group). DG: Dentate Gyrus. Scale bar for immunohistochemical images is 200 μm. *p<0.05; **p<0.01.

We found a significant reduction in *Il-6* and *Tnf-α*, as well as a slight but not significant decrease in *C-X-C motif chemokine ligand 10* *(Cxcl10)* gene expression between 5XFAD treated with UNC0642 compared to 5XFAD Control mice group (Figure 4A). Likewise, a significant reduction in *Il-6, Tnf-α,* and *Cxcl10* between 5XFAD Control in comparison with Wt Control was found (Figure 4A). Moreover, no changes in *Monocyte chemoattractant protein 1* (*Mcp1)* gene expression among mice groups were observed (Figure 4A).

On the other hand, immunostaining quantification of Glial fibrillary acidic protein (GFAP) fluorescence intensity revealed that UNC0642 treatment reduced GFAP staining, especially in the Dentate Gyrus (DG) and CA3 regions, reaching it to Wt Control (Figure 4B–4E). However, no changes in the immunostaining quantification of Ionized calcium binding adapter molecule 1 (IBA1) between 5XFAD treated with UNC0642 and 5XFAD Control were found (Figure 4F–4I), whereas a significant reduction of IBA1 immunostaining quantification in Wt Control in comparison with 5XFAD Control was determined (Figure 4F–4I).

### Increased synaptic marker and neurotrophins induced by UNC0642 in 5XFAD mice

**Figure 5:**
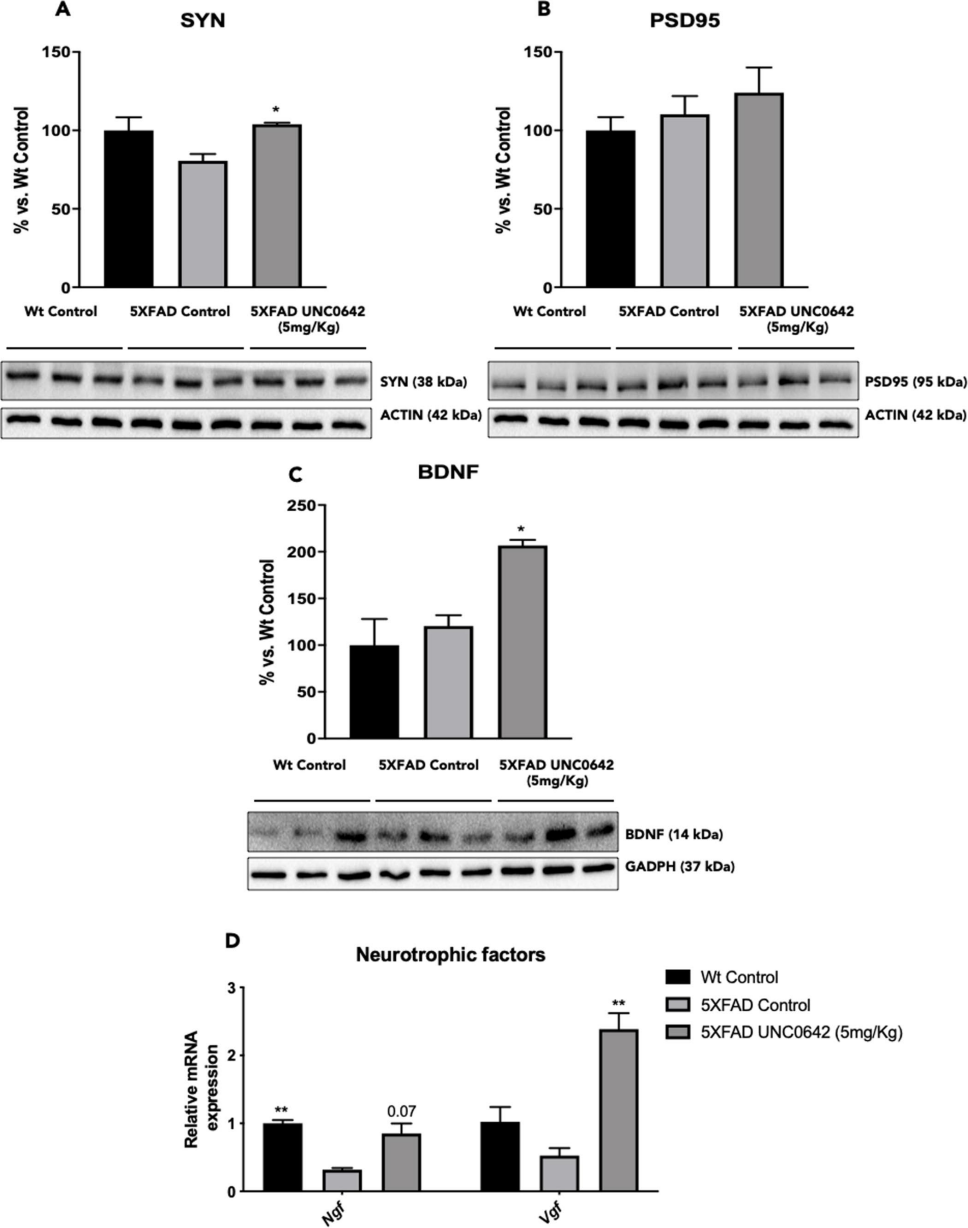
Representative WB, and quantification of neuroplasticity markers for SYN (**A**), PSD95 (**B**), and BDNF (**C**). Representative gene expression of neurotrophic factors for *Ngf,* and *Vgf* (**D**). Values in bar graphs are adjusted to 100% for protein levels of the Wt Control. Gene expression levels were determined by real-time PCR. Values represented are mean ± Standard error of the mean (SEM); (n = 4-6 for each group). *p<0.05; **p<0.01.

A significant increase in Synaptophysin (SYN) protein levels was found in 5XFAD treated with UNC0642 compared to the 5XFAD Control group, reaching Wt Control levels (Figure 5A). Albeit did not reach significance, UNC0642 treatment increased Postsynaptic density protein 95 (PSD95) protein levels (Figure 5B).

In addition, we evaluated the BDNF another target of G9a/GLP, and we found a significant increase in BDNF protein levels in 5XFAD treated with UNC0642 in comparison with the 5XFAD Control mice group (Figure 5C). Next, we determined the gene expression of neurotrophic factors such as *Nerve growth factor* *(Ngf)*, and *Nerve growth factor inducible* *(Vgf)*. We found restored gene expression, for both of them, in 5XFAD treated with UNC0642 in comparison with 5XFAD Control, being only significant in *Vgf* gene (Figure 5D).

### UNC0642 treatment reduced β-amyloid plaques in 5XFAD mice

**Figure 6:**
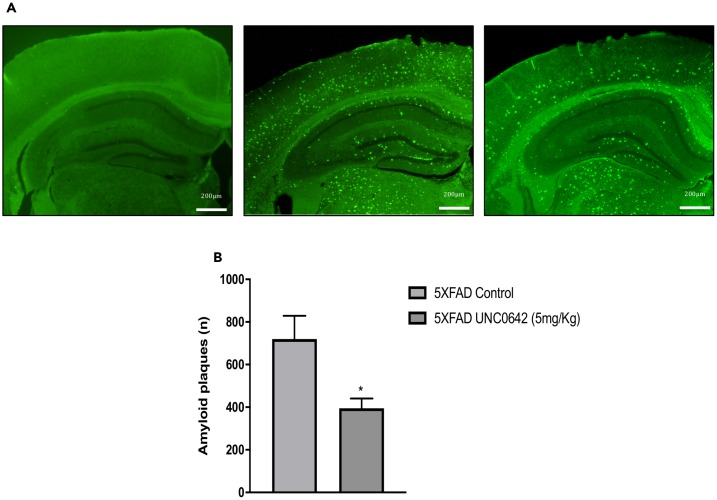
Representative images (**A**) and quantifications (**B**) of β-amyloid plaques stained with Thioflavin-S in Wt Control, 5XFAD Control and 5XFAD treated with UNC0642 (5mg/Kg). Values represented are mean ± Standard error of the mean (SEM); (n = 4 for each group). *p<0.05.

**Figure 7:**
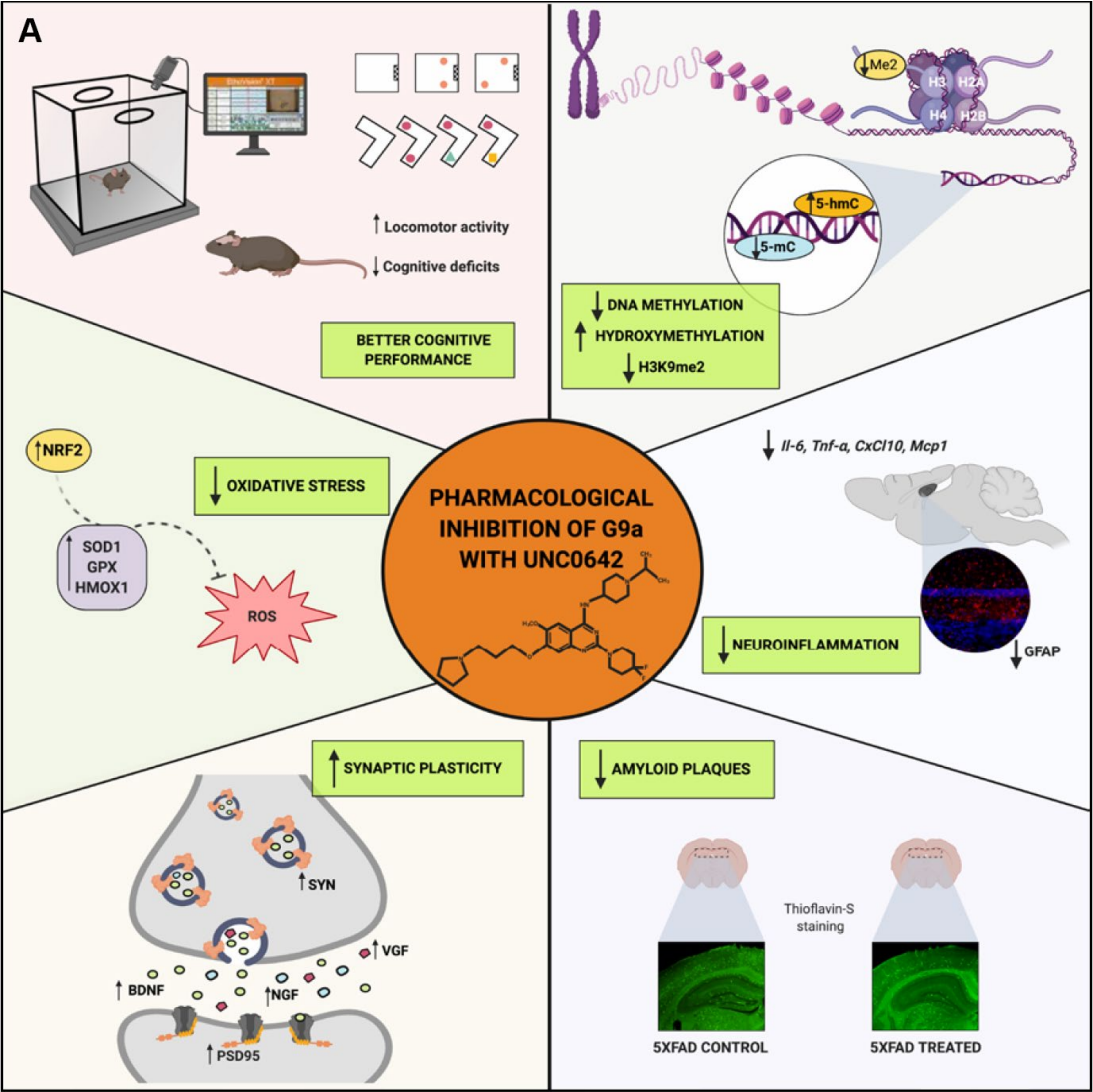
Scheme of epigenetic and molecular mechanisms changed in 5XFAD mice induced by pharmacological inhibition of G9a/GLP by UNC0642 “Created with BioRender.com” (**A**).

Finally, we evaluated the effect of UNC0642 treatment on β-amyloid pathology in 5XFAD mice. The pharmacological inhibition of G9a/GLP had a strong effect in reducing the number of β-amyloid plaques stained with Thioflavin-S (by an average of 45%) (Figure 6A and 6B), indicating the prevention of β-amyloid burden in a transgenic mouse model characterized by rapidly develops β-amyloid plaques pathology. An overview of the effect of inhibiting the G9a/GLP complex with UNC0642 in the 5XFAD mouse model can be seen in (Figure 7).

## DISCUSSION

It is well established that epigenetic modifications are associated with neurodegeneration and cognitive decline. Because of the deregulation of transcriptional activity, resulting in an aberrant neuronal function [36, 37]. Those epigenetic alterations can be the consequence of mutations in genes coding for proteins directly involved in core processes of methylation and histone modification [38]. Thus, much research has focused on rescuing the cognitive deficit through epigenetic-based therapies during AD to normalize the epigenetic profile and the associated cognitive decline. For instance, the regulation of the histone acetylation in *in vitro* studies and AD mouse models has been demonstrated useful [33, 39–41]. To this line epigenetic regulation by G9a/GLP histone lysine–methyltransferase complex is emerging as a critical mechanism underlying the learning and memory processes [42–44]. In fact, H3K9me2 (a mark associated with gene repression) and the H3K9-specific histone methyltransferase G9a have been linked to memory consolidation [23, 44, 45].

The present study investigated the neuroprotective effects of UNC0642, a potent and specific G9a/GLP inhibitor in 5XFAD mice model. Cognitive performance and several molecular pathways regulated by this chromatin-modifying enzyme were studied. The pharmacological intervention was applied at the age of 8-month-old when cognitive impairment correlates with β-amyloid plaques deposition and epigenetic alterations in 5XFAD mice model [33].

Here, we demonstrated that pharmacological inhibition of G9a/GLP displayed better behavioural task, showing more locomotor activity and less anxiety-behaviour, as well as showed improvement in recognition and spatial memory in 5XFAD mice. In the same line, it has been reported that chronic treatment with UNC0642 and A-366 decreased anxiety-like behaviours in adult male mice [27]. Furthermore, in another study, it has been found that treatment of 5XFAD mice with G9a/GLP inhibitors leads to the remarkable restoration of the cognition [46]. Accordingly, the cognitive improvement achieved by UNC0642 treatment in 5XFAD mice parallels with the histone methylation mark H3K9me2, and with global epigenetic marks 5-mC and 5-hmC.

Recent studies have suggested that aberrant 5-mC and 5-hmC is associated with neurodegeneration and AD through altering the structure of the DNA double helix that affects gene expression [47]. In addition, these epigenetic marks are essential for synaptic plasticity [45], cognitive function [48], and age-related alterations [37]. We have previously described that 5XFAD presented a higher degree of global 5-mC and a diminution in 5-hmC, which paralleled with Aβ deposition [33]. Here, we found reduced global 5-mC and increased 5-hmC levels in 5XFAD mice treated with UNC0642. In the same line, non-pharmacological interventions like Environmental Enrichment (EE) improved cognition in rodents after changes in those epigenetic marks after [49]. A reduction in H3K9me2 levels in the 5XFAD mice hippocampus treated with UNC0642. Concretely, UNC0642 reduced the transcriptional silencing of this mark that demonstrate the direct mechanistic cause for the cognitive improvement observed. Consistent with our results, few studies have found significantly elevated levels of H3K9me2, which correlates with synaptic dysfunction and cognitive impairment in mice and AD human post-mortem tissues [46]. Besides, those works showed that the treatment with the G9a/GLP inhibitor brought down H3K9me2 levels in the brain.

Besides cognitive and epigenetic improvement induced by UN0642 in 5XFAD mice, molecular and biochemical pathways modulated by G9a/GLP were studied. It is well accepted that OS as a consequence of ROS accumulation is associated with neurodegeneration [50]. OS occurs when the balance between antioxidant enzymes and ROS are disrupted [51, 52] and can influence several cellular pathways, from DNA and histone to histone chromatin-modifying enzymes, which directly affect the epigenetic landscape [53]. Of interest, decreased protein levels of the NRF2 have been reported in AD [54]. Therefore, overexpression of antioxidant enzymes may confer protection against oxidative insults [55]. By last, recently, it has been demonstrated that an administration of G9a/GLP inhibitor attenuates the induction of H3K9me2, activating NRF2 and reducing OS [56]. When we evaluated the NRF2 pathway, *Hmox1* gene expression was significantly increased, although increases in SOD1 and GPX1 protein did not reach significance. However, the H_2_O_2_ concentration was significantly reduced, demonstrating OS reduction in 5XFAD mice treated with UNC0642.

Neuroinflammation has a key role in neurodegenerative disorders, including AD [57]. Furthermore, there is evidence suggesting that epigenetic mechanisms may lead to inflammation by modulating the expression of pro-inflammatory cytokines [58]. In this study, we found that the inhibition of G9a/GLP by UNC0642 reduced gene expression of *Il-6, Tnf-α*, and *Cxcl10.* GFAP, a marker of astrogliosis, was drastically reduced. Conversely, IBA1 levels were not modified. It is well nown that there is a differential relation of reactive astrocytes and microglial activation to the β-amyloid plaques in AD [59]. Furthermore, whereas microglial activation can play a role in plaque removal, the astrogliosis has been related to proinflammatory state [60, 61]. Therefore, our findings agree with several reports describing that G9a-dependent H3K9me2 is associated with several inflammatory pathways, including T cell receptor signalling, Interleukin 4 (IL-4) signalling, and GATA3 transcription [22]. Likewise, inhibition of G9a/GLP activity with the small-molecule inhibitors BIX-01294 or UNC0638 resulted in enhanced T cell differentiation, reducing inflammation [62].

One of the critical early events in AD is the loss of synaptic plasticity because [63]. These synaptic failures are affected by the Aβ accumulation as well as epigenetic marks such as CpG methylation and histone modifications [64]. Of interest, several studies demonstrated that G9a and/or GLP inhibition leads to *Bdnf* upregulation expression and them neuroprotection in different conditions, such as in a model of hypoxic metabolic stress [65], or in hippocampal slices of CA1 region from male Wistar rats [17]. UNC0642 treatment increased synaptic markers, as SYN, and neurotrophic factors such as *Ngf, Vgf*, and BDNF in 5XFAD, demonstrating that this epigenetic target is also working on the neuroplasticity.

Finally, UNC0642 reduced Aβ burden, the main neuropathological AD hallmark in 5XFAD brain. Although several studies have previously demonstrated the *in vivo* neuroprotective properties of the G9a/GLP inhibition [26], this is the first *in vivo* studies, which assess a reduction in the Aβ burden.

Taken together, we demonstrated that G9a/GLP inhibition with UNC0642 has neuroprotective effects in a transgenic mouse model of EOAD, improving cognitive performance through reduction in its repressive chromatin mark H3K9me2 and changing the global levels of 5-mC and 5-hmC. Noteworthy, UNC0642 prevented Aβ plaques accumulation, increased synaptic plasticity and neuronal markers that are characteristically loss in AD. Moreover, UNC0642 was able to reduce OS and neuroinflammation. Thus, our results provide new evidence that inhibition G9a/GLP activity might be a promising target for AD therapy (Figure 7).

## MATERIALS AND METHODS

### Animals

Male Wt (n = 10), 5XFAD (n = 17) (8-month-old) were used to perform cognitive and molecular studies. We divided these mice randomly into three groups: Wt Control (n = 10), 5XFAD Control (n = 10), and 5XFAD treated with G9a/GLP histone methyltransferase inhibitor, the UNC0642 (5XFAD UNC0642 (5mg/Kg) n = 7). The sample size for the intervention was chosen following previous studies in our laboratory and using one of the available interactive tool (http://www.biomath.info/ power/index.html). Animals had free access to food and water and were kept under standard temperature conditions (22±2°C) and 12h: 12h light-dark cycles (300 lux/0 lux). UNC0642 (5mg/Kg/day) was dissolved in 1,8% 2-hydroxypropyl-β-cyclodextrin and administered through drinking water for 4 weeks. After the treatment period, behavioural tests were performed in the animals. Water consumption was controlled each week, and UNC0642 concentration was adjusted accordingly to reach the optimal dose.

Studies and procedures involving mouse behaviour test, brain dissection and extractions followed the ARRIVE and standard ethical guidelines (European Communities Council Directive 2010/63/EU and Guidelines for the Care and Use of Mammals in Neuroscience and Behavioural Research, National Research Council 2003) and were approved by Bioethical Committees from University of Barcelona and Government of Catalonia. All efforts were made to minimize the number of animals used and their suffering.

### Behavioural tests

### Open field test

The OFT was performed as previously described [66]. The floor was divided into two areas defined as the center and peripheral zone. Behaviour was scored with SMART® ver.3.0 software, and each trial was recorded for later analysis using a camera situated above the apparatus. Mice were placed at the center and allowed to explore the white polywood box (50x50x25 cm) for 5 minutes. Afterward, the mice were returned to their home cages, and the OFT apparatus was cleaned with 70% ethanol (EtOH). The parameters scored included center staying duration, rearings, defecations, and the distance travelled, calculated as the sum of global distance travelled in the open field arena for 5 minutes.

### Novel object recognition test

The NORT protocol employed was a modification of [67, 68]. Briefly, mice were placed in a 90°, two-arm, 25-cm-long, 20-cm-high, 5-cm-wide black maze. Before performing the test, the mice were individually habituated to the apparatus for 10 minutes for 3 days. On day 4, the animals were allowed to explore freely a 10 minutes acquisition trial (First trial), during which they were placed in the maze in the presence of two identical, novel objects at the end of each arm. After a delay (2h and 24h), the animal was allowed to explore two objects one old object and one novel object. The time that mice explored the Novel object (TN) and Time that mice explored the Old object (TO) were measured. A DI was defined as (TN−TO)/(TN+TO). Exploration of an object was defined as pointing the nose towards at a distance ≤2 cms and/or touching it with the nose. Turning or sitting around the object was not considered exploration. In order to avoid object preference biases, objects were counterbalanced.

### Object location test

The OLT is a well-established task based on the spontaneous tendency of rodents to spend more time exploring a novel object location than a familiar object location, as well as to recognize when an object has been relocated [69]. Briefly, the test was performed during 3 days in a wooden box (50 × 50 × 25 cm), in which three walls were white except one that was black. The first day, the box was empty, and the animals just habituated to the open field arena for 10 minutes. The second day, two objects were placed in front of the black wall, equidistant from each other and the wall. The objects were 10-cm high and identical. The animals were placed into the open field arena and allowed to explore both objects and surroundings, for 10 minutes. Afterward, animals were returned to their home cages, and the OLT apparatus was cleaned with 70% EtOH. The third day, one object was moved in front of the white wall to test the spatial memory. Trials were recorded using a camera mounted above the open field area, and the total exploration time was determined by scoring the amount of time (seconds) spent sniffing the object in the new location (TN) and the object in the old location (TO). In order to evaluate the cognitive performance, the DI was calculated, which is defined as (TN-TO)/(TN+TO).

### Immunodetection experiments

### Brain processing

Mice were euthanized by cervical dislocation one day after the cognitive tests finished. Brains were immediately removed from the skull. The hippocampus was then isolated and frozen in powdered dry ice. They were maintained at −80°C until protein extraction, RNA and, DNA isolation. For protein extraction, tissue samples were homogenized in lysis buffer containing phosphatase and protease inhibitors (Cocktail II, Sigma-Aldrich). Total protein levels were obtained, and protein concentration was determined by the method of Bradford.

### Protein levels determination by Western blotting

For WB, aliquots of 15 μg of hippocampal protein were used. Protein samples from 12 mice (n = 4 per group) were separated by Sodium dodecyl sulphate-Polyacrylamide gel electrophoresis (SDS-PAGE) (8-12%) and transferred onto (Polyvinylidene difluoride) PVDF membranes (Millipore). Afterward, membranes were blocked in 5% non-fat milk in 0,1% Tris-buffered saline - Tween20 (TBS-T) for 1 hour at room temperature, followed by overnight incubation at 4°C with the primary antibodies listed in Table 2.

**Table 2:** Antibodies used in Western blot studies.

**Antibody**	**Host**	**Source/Catalog**	**WB dilution**
SOD1	Sheep	Calbiochem/574597	1:1000
SYN	Rabbit	Dako/CloneSY38	1:2000
PSD95	Rabbit	Abcam/ab18258	1:1000
H3K9me2	Rabbit	Epigentek/A-4035	1:1000
GPX1	Rabbit	Novus Biological/NBP1-33620	1:1000
BDNF	Rabbit	Bios/BS-4989R	1:1000
NRF2	Rabbit	Cell Signaling/DIZ9C	1:1000
TBP	Mouse	Abcam/ab51841	1:1000
Actin	Mouse	Sigma-Aldrich/A5441	1:2000
GAPDH	Mouse	Millipore/MAB374	1:5000
Goat-anti-mouse HRP conjugated		Biorad/170-5047	1:2000
Goat-anti-rabbit HRP conjugated		Biorad/170-6515	1:2000
Rabbit-anti-sheep HRP conjugated		Abcam/ab97130	1:2000

Membranes were washed and incubated with secondary antibodies for 1 hour at room temperature. Immunoreactive proteins were viewed with a chemiluminescence-based detection kit, following the manufacturer's protocol (ECL Kit; Millipore) and digital images were acquired using a ChemiDoc XRS+ System (BioRad). Semi-quantitative analyses were carried out using ImageLab software (BioRad), and results were expressed in Arbitrary Units (AU), considering control protein levels as 100%. Protein loading was routinely monitored by immunodetection of glyceraldehyde-3-phosphate dehydrogenase (GADPH) or β-actin.

### Immunofluorescence

Coronal section of 30 μm was obtained by a cryostat (Leica Microsystems CM 3050S, Wetzlar, Germany) and kept in a cryoprotectant solution at −20°C.

First, free-floating slices were selected and placed on a 24-wells plaque. After that, were washed five times with PBS 0.01M + 1% Triton X-100. Then, free-floating sections were blocked with a solution containing 5% fetal bovine serum (FBS), 1% Triton X-100, PBS 0.01M + gelatine 0.2% for 2h at room temperature. Afterward, slices were washed with PBST (PBS 0.1M, 1% Triton X-100) five times for 5 minutes each and were incubated with the primary antibodies listed in Table 3, over-night at 4°C. On the following day, coronal slices were washed with PBST 6 times for 5 minutes and then incubated with the secondary antibodies at room temperature for 2h. Later, sections were co-incubated with, 1mg/ml DAPI staining solution (Sigma-Aldrich, St. Louis, MI) for 5 minutes in the dark at room temperature and washed with PBS 0.01M. Finally, the slices were mounted using Fluoromount G (EMS, USA) and image acquisition was performed with a fluorescence laser microscope (Olympus BX51, Germany). At least 3 images from 4 different individuals by the group were analyzed with ImageJ/Fiji software available online from the National Institutes of Health.

**Table 3:** Antibodies used in Immunofluorescence studies.

**Antibody**	**Host**	**Source/Catalog**	**WB dilution**
GFAP	Rabbit	Abcam/ab48050	1:1000
IBA1	Rabbit	Abcam/ab16589	1:1000
Alexa Fluor® 594	Goat	TermoFisher/ab150080	1:400

### β-amyloid plaques histology

β-amyloid plaques from 12 mice (n = 4 per group) were stained with Thioflavin-S. The frozen brains were embedded into OCT Cryostat Embedding Compound (Tissue-Tek, Torrance, CA, USA) and then cut into 30 μm- thick sections at −20 °C using a cryostat (Leica Microsystems, Germany) and kept in a cryoprotectant solution at −20°C. Free-floating slices were selected and placed on a 24-wells plaque. For the Thioflavin-S staining procedure, the brain sections were first rehydrated at room temperature by 5 minutes incubation in PBS. To continue with, brain sections were incubated with 0.3% Thioflavin-S (Sigma-Aldrich) solution for 20 minutes at room temperature in the dark. Subsequently, these samples were submitted to washes in 3 minutes series, specifically with two washes using 80% EtOH, one wash using 90% ethanol and three washes with PBS. Then, slides were mounted with Fluoromount-GTM (EMS, Hatfield, NJ, USA) and allowed to dry overnight. Image acquisition was performed with a fluorescence laser microscope (Olympus BX51, Germany). For plaque quantification, similar and comparable histological areas were selected, focusing on the adjacent positioning of the whole cortical area and the hippocampus.

### Determination of OS in the hippocampus

Hydrogen peroxide from 9 mice (n = 3 per group) was measured as an indicator of OS, and it was quantified using the Hydrogen Peroxide Assay Kit (Sigma-Aldrich, St. Louis, MI) according to the manufacturer’s instructions.

### Global DNA methylation and hydroxymethylation quantification

Isolation of genomic DNA from 12 samples (n = 4 per group) was conducted using the FitAmpTM Blood and Cultured Cell DNA Extraction Kit, according to the manufacturer's instructions. Then, Methylflash Methylated DNA Quantification Kit (Epigentek, Farmingdale, NY, USA) and MethylFlash HydroxyMethylated DNA Quantification Kit were used in order to detect methylated and hydroxymethylated DNA. Briefly, these kits are based on specific antibody detection of 5-mC and 5-hmC residues, which trigger an Enzyme-Linked Immunosorbent Assay (ELISA)-like reaction that allows colorimetric quantification by reading absorbance at 450 nm using a Microplate Photometer. The absolute amount of methylated or hydroxymethylated DNA (proportional to the Optical Density [OD] intensity) was measured and quantified using a standard curve plotting OD values vs. five serial dilutions of a control methylated and hydroxymethylated DNA (0.5–10 ng).

### RNA extraction and gene expression determination

Total RNA isolation was carried out using Trizol reagent following the manufacturer’s instructions. The RNA content in the samples was measured at 260 nm, and the purity of the samples was determined by the A260/280 and A260/230 ratio in a NanoDrop^™^ ND-1000 (Thermo Scientific). Reverse transcription-Polymerase Chain Reaction (RT-PCR) was performed as follows: 2 μg of messenger RNA (mRNA) was reverse-transcribed using the High Capacity cDNA Reverse Transcription kit (Applied Biosystems). Real-time quantitative PCR (qPCR) was employed to quantify the mRNA expression of a set of OS, inflammatory markers, and neurotrophic factors listed in Table 3.

SYBR® Green real-time PCR was performed on a Step One Plus Detection System (Applied-Biosystems) employing SYBR® Green PCR Master Mix (Applied-Biosystems). Each reaction mixture contained 6.75 μL of complementary DNA (cDNA) (which concentration was 2 μg), 0.75 μL of each primer (which concentration was 100 nM), and 6.75 μL of SYBR® Green PCR Master Mix (2X).

TaqMan-based real-time PCR (Applied Biosystems) was also performed in a Step One Plus Detection System (Applied-Biosystems). Each 20 μL of TaqMan reaction contained 9 μL of cDNA (18 ng), 1 μL 20X probe of TaqMan Gene Expression Assays and 10 μL of 2X TaqMan Universal PCR Master Mix.

Data were analyzed using the comparative Cycle threshold (Ct) method (ΔΔCt), where the housekeeping gene level was used to normalize differences in sample loading and preparation [33]. Normalization of expression levels was performed with *β-actin* for SYBR® Green-based real-time PCR results and TATA-binding protein *(Tbp)* for TaqMan-based real-time PCR. Primers and TaqMan probes are listed in Table 4. Each sample (n = 6 per group) was analyzed in duplicate, and the results represent the n-fold difference of the transcript levels among different groups.

**Table 4:** Primers and probes used in qPCR studies.

**SYBR Green primers**			
**Target**	**Product size (bp)**	**Forward primer (5′-3′)**	**Reverse primer (5′-3′)**
***Il-6***	189	ATCCAGTTGCCTTCTTGGGACTGA	TAAGCCTCCGACTTGTGAAGTGGT
***Tnf-α***	157	TCGGGGTGATCGGTCCCCAA	TGGTTTGCTACGACGTGGGCT
***Cxcl10***	72	GGCTAGTCCTAATTGCCCTTGG	TTGTCTCAGGACCATGGCTTG
***Mcp1***	159	CCCACTCACCTGCTGCTACT	TCTGGACCCATTCCTTCTTG
***Ngf***	111	GGAGCGCATCGAGTGACTT	CCTCACTGCGGCCAGTATAG
***Vgf***	178	GTCAGACCCATAGCCTCCC	CTCGGACTGAAATCTCGAAGTTC
***β-actin***	190	CAACGAGCGGTTCCGAT	GCCACAGGTTCCATACCCA
**Taqman probes**			
**Target**		**Product size (bp)**	**Reference**
***Hmox1***		69	Mm00516005_m1
***Tbp***		93	Mm00446971_m1

### Data analysis

Data analysis was conducted using GraphPad Prism ver. 8 statistical software. Data are expressed as the mean ± Standard error of the mean (SEM) of at least 3 samples per group. Means were compared with One-way Analysis of variance (ANOVA), followed by the Dunnett post hoc test. Comparison between groups was also performed by two-tailed Student’s t-test for independent samples when it was necessary. Statistical significance was considered when p values were <0.05. The statistical outliers were determined with Grubs' test and when necessary were removed from the analysis.
